# Differentiation of Human Tonsil-Derived Mesenchymal Stem Cells into Schwann-Like Cells Improves Neuromuscular Function in a Mouse Model of Charcot-Marie-Tooth Disease Type 1A

**DOI:** 10.3390/ijms19082393

**Published:** 2018-08-14

**Authors:** Saeyoung Park, Namhee Jung, Seoha Myung, Yoonyoung Choi, Ki Wha Chung, Byung-Ok Choi, Sung-Chul Jung

**Affiliations:** 1 Department of Biochemistry, College of Medicine, Ewha Womans University, Seoul 07985, Korea; saeyoung@ewha.ac.kr (S.P.); polypola@ewhain.net (N.J.); myothink@naver.com (S.M.); yychoi27@ewhain.net (Y.C.); 2 Department of Biological Sciences, Kongju National University, Gongju 32588, Korea; kwchung@kongju.ac.kr; 3 Department of Neurology, Samsung Medical Center, Sungkyunkwan University School of Medicine, Seoul 06351, Korea; bochoi77@hanmail.net

**Keywords:** tonsil-derived mesenchymal stem cells, Schwann cells, Charcot-Marie-Tooth disease type 1A, remyelination, neuromuscular regeneration

## Abstract

Charcot-Marie-Tooth disease type 1A (CMT1A) is the most common inherited motor and sensory neuropathy, and is caused by duplication of *PMP22*, alterations of which are a characteristic feature of demyelination. The clinical phenotype of CMT1A is determined by the degree of axonal loss, and patients suffer from progressive muscle weakness and impaired sensation. Therefore, we investigated the potential of Schwann-like cells differentiated from human tonsil-derived stem cells (T-MSCs) for use in neuromuscular regeneration in trembler-J (Tr-J) mice, a model of CMT1A. After differentiation, we confirmed the increased expression of Schwann cell (SC) markers, including glial fibrillary acidic protein (GFAP), nerve growth factor receptor (NGFR), S100 calcium-binding protein B (S100B), glial cell-derived neurotrophic factor (GDNF), and brain-derived neurotrophic factor (BDNF), which suggests the differentiation of T-MSCs into SCs (T-MSC-SCs). To test their functional efficiency, the T-MSC-SCs were transplanted into the caudal thigh muscle of Tr-J mice. Recipients’ improved locomotive activity on a rotarod test, and their sciatic function index, which suggests that transplanted T-MSC-SCs ameliorated demyelination and atrophy of nerve and muscle in Tr-J mice. Histological and molecular analyses showed the possibility of in situ remyelination by T-MSC-SCs transplantation. These findings demonstrate that the transplantation of heterologous T-MSC-SCs induced neuromuscular regeneration in mice and suggest they could be useful for the therapeutic treatment of patients with CMT1A disease.

## 1. Introduction

Charcot-Marie-Tooth (CMT) disease results from inherited neuropathies caused by over 50 different mutation genes [1], and the type of disease is classified according to those genes. Type 1A is the most common type, caused by a mutation in the gene encoding peripheral myelin protein 22 (PMP22) resulting in altered gene expression and structural defects. Axonal dysfunction in peripheral nerves is a common feature of the CMT disease type 1A (CMT1A) forms, resulting in muscle weakness, gait abnormalities, and foot deformities, for which there are no pharmacological treatments [2,3,4]. A point mutation, L16P (leucine 16 to proline), in PMP22 underlies a form of human CMT1A, and also underlies the CMT1A phenotype in mice. We used Trembler-J (Tr-J) mice in this study, which harbor this mutation in *pmp22* [5].

Peripheral nerve regeneration is a complicated process characterized by Wallerian degeneration, axonal sprouting, and remyelination. Schwann cells (SCs) are glial cells of peripheral nerves that wrap around axons to form myelin in the peripheral nervous system and play an integral role in multiple facets of nerve regeneration. SC transplantation as a cell-based therapy is limited by the invasive nature of harvesting and donor site morbidity. However, stem-cell transplantation can avoid these limitations and bring benefit to the process of peripheral nerve regeneration [6]. Various types of stem cells, such as embryonic stem cells [7,8], induced pluripotent stem cells [9], neural stem cells [10], bone marrow-derived stem cells (BM-MSCs) [11,12], adipose-derived stem cells (Ad-MSCs) [13,14], amniotic tissue-derived stem cells [15], amniotic fluid-derived stem cells [16], and umbilical cord-derived MSCs (UC-MSCs) [17] have been reported to help peripheral nerve regeneration.

T-MSCs present typical features of MSCs, including the ability to differentiate into tissues of the three primary germ layers, with no adverse effects of long-term culture (over 15 passages) or cryopreservation [18,19,20,21,22]. T-MSCs are useful for stem-cell therapy in various disease conditions because tonsils are a ready source of stem cells [23,24]. T-MSCs have the potential to differentiate into Schwann-like cells and can secrete neurotrophic factors to promote axonal growth and remyelination [18].

In the present study, we assessed the potential of T-MSC-derived SCs (T-MSC-SCs) as a cell therapy for peripheral nerve regeneration in the Tr-J mouse model of CMT1A disease. After the transplantation of T-MSC-SCs into the muscle adjacent to the sciatic nerve, the effect of overcoming demyelination, which is the most fundamental cause of CMT1A disease, and their therapeutic effects on axons and muscles were investigated.

## 2. Results

### 2.1. T-MSC-Derived SCs (T-MSC-SCs) Exhibit Schwann Cell and Neurotrophic Markers

**Figure 1:**
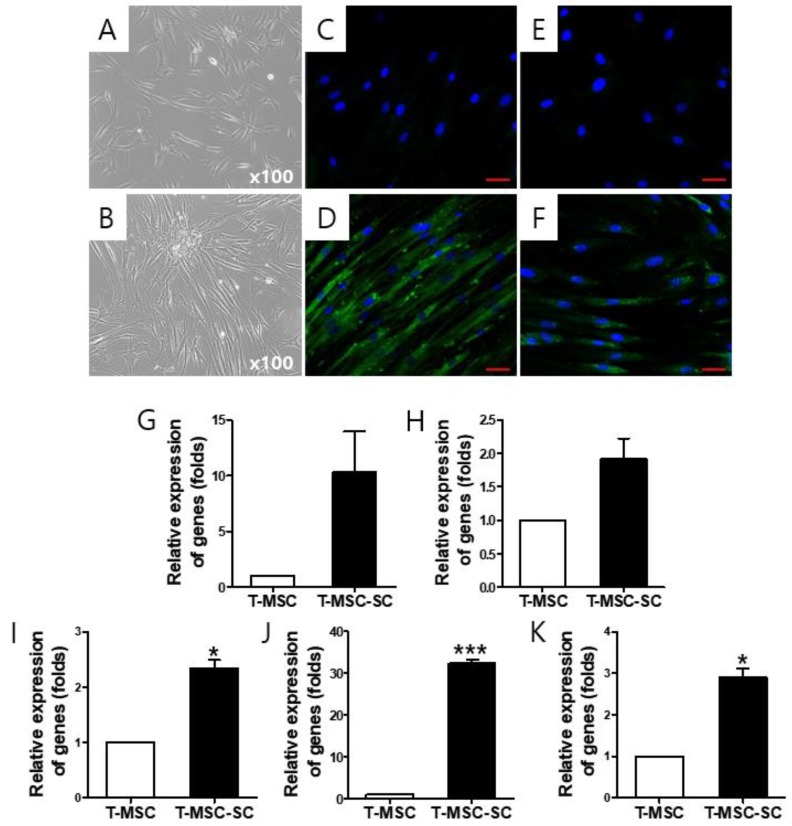
The differentiation potential of tonsil-derived mesenchymal stem cells (T-MSCs) toward Schwann cells (SCs). (**A**) Undifferentiated T-MSCs were induced to form SCs. (**B**) T-MSC-SCs were cultured for 16 days in SC differentiation medium. Original magnification, ×100. Immunostaining for glial fibrillary acidic protein (GFAP) (**C**,**D**: blue, 4′,6-diamidino-2-phenylindole (DAPI); green, GFAP) and nerve growth factor receptor (NGFR) (**E**,**F**: blue, DAPI; green, NGFR) expression levels were compared before and after SC induction. Representative images of the differentiation potential show GFAP (**D**) or NGFR (**F**) staining on the TMSC-SC groups compared with the T-MSC group (**C**,**E**). Expression of SCs (**G**: *GFPA*; **H**: *NGFR;* **I**: S100 calcium-binding protein B (*S100B*)) and neurotrophic (**J**: glial cell-derived neurotrophic factor (GDNF)); (**K**: brain-derived neurotrophic factor (BDNF)) markers in these cells were examined using real-time qPCR to determine the differentiation of SCs. The mRNA was isolated from undifferentiated T-MSCs and T-MSC-SCs, and expression levels were normalized against the expression of the housekeeping gene encoding glyceraldehyde 3-phosphate dehydrogenase (*GAPDH*). The results are reported as ratios of the marker gene expression of T-MSC-SCs versus undifferentiated T-MSCs. Data are the means ± SEMs of experiments performed in triplicate. * *p* < 0.05; *** *p* < 0.001. Scale bars = 50 μm.

To assess their potential for neuromuscular regeneration in Tr-J mice in vivo, we transplanted T-MSC-SCs. The T-MSCs (Figure 1A,C,E) were cultured for 16 days to allow their terminal differentiation into T-MSC-SCs (Figure 1B,D,F), when they then displayed elongated bi- or tripolar spindle-shaped morphology and thinner cytoplasmic extensions, as previously reported [18]. To determine the phenotypes of the T-MSC-SCs, we examined for SC and neurotrophic markers, such as glial fibrillary acidic protein (GFAP), S100 calcium-binding protein B (S100B), nerve growth factor receptor (NGFR), glial cell-derived neurotrophic factor (GDNF), and brain-derived neurotrophic factor (BDNF) using immunostaining (Figure 1C–F) and real-time PCR (Figure 1G–K). The T-MSC-SCs exhibited increased expression of these markers of differentiation compared with undifferentiated T-MSCs. The ratio of NGFR-positive cells was 69.7 ± 7.6%.

### 2.2. Motor Function after Transplanting T-MSC-SCs into the Tr-J Mice

**Figure 2:**
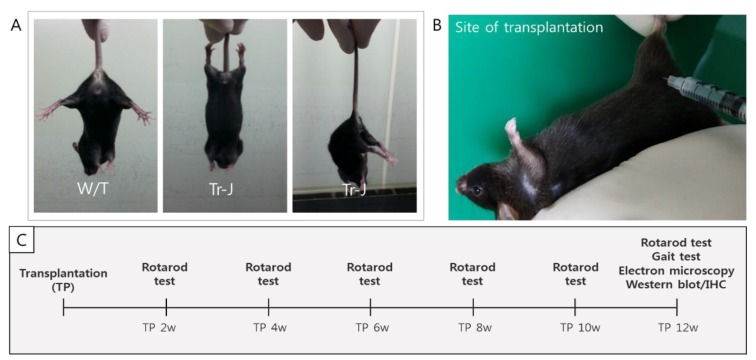
Features of Tr-J mice as a model of CMT1A disease and plan for transplanting T-MSC-SCs into mice. (**A**) Representative image of the body position of wild-type (W/T) mice and heterozygous Tr-J mice during a tail suspension test; (**B**) The transplanted site at the right thigh muscle near the sciatic nerve of Tr-J/^+^ mice; (**C**) Schematic of experiments.

**Figure 3:**
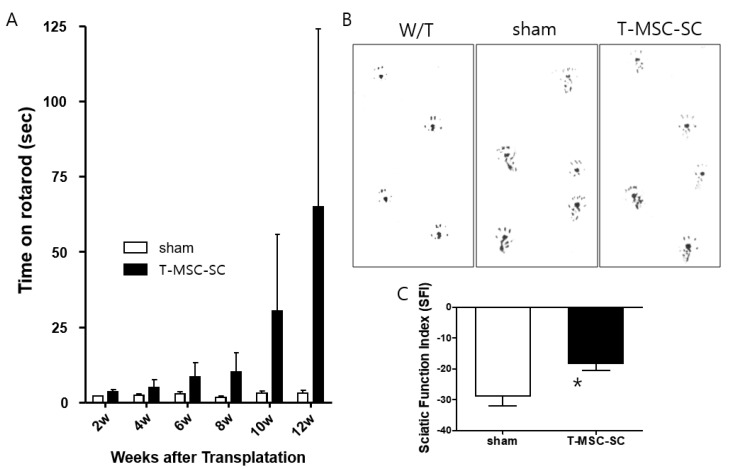
Assessment of motor function in Tr-J mice following transplantation with T-MSC-SCs using a rotarod test and footprints. (**A**) Latencies in the T-MSC-SC-recipient mice improved gradually by 12 weeks after transplantation. Open bars indicate the latencies of animals injected with phosphate-buffered saline (PBS) (sham group; *n* = 7); filled bars indicate those of the T-MSC-SCs recipient animals (T-MSC-SCs group; *n* = 7); (**B**) Representative image of footprint during the gait test of wild-type mice (panel W/T), Tr-J mice (panel sham), and mice in the T-MSC-SCs group (panel T-MSC-SC); (**C**) The sciatic function index (SFI) from footprinting analysis 12 weeks after transplantation (*n* = 7 for each group, * *p* < 0.05).

After transplanting T-MSC-SCs and/or injecting phosphate-buffered saline (PBS) (sham treatment) into the right thigh muscle near the sciatic nerve of the Tr-J mice and we assessed their phenotype. Using a rotarod test, we observed improvement in motor function in the T-MSC-SC group at 2, 4, 6, 8, 10, and 12 weeks (Figure 2). The latencies of mice in the T-MSC-SC group (*n* = 7) on a rotating rod elevated gradually by 12 weeks, but no improvement was observed in the sham-treatment group (*n* = 7) animals. No significant differences in the results of rotarod tests were found between any group (Figure 3A). The latency of the age-matched wild-type (W/T) group (*n* = 8) was 400 s in this study. The T-MSC-SC-recipient mice were able to stand with their front limbs resting on a wall, which was not seen in the sham group (Figure S1). For functional assessment of regeneration effected by transplanted T-MSC-SCs in Tr-J mice, the sciatic function index (SFI, Figure 3C) was calculated at 12 weeks after transplantation using footprint patterns (Figure 3B). In general, the SFI fluctuates around 0 for normal nerve (W/T), whereas it is −28.79 ± 3.214 in the sham group, where SFI represents dysfunction. The SFI of the T-MSC-SC group (–18.25 ± 2.244) revealed a significant improvement compared with the sham group (*p* < 0.05). SFI was negative; a higher SFI indicates better functioning of the sciatic nerve.

### 2.3. Ultrastructure of the Sciatic Nerve

**Figure 4:**
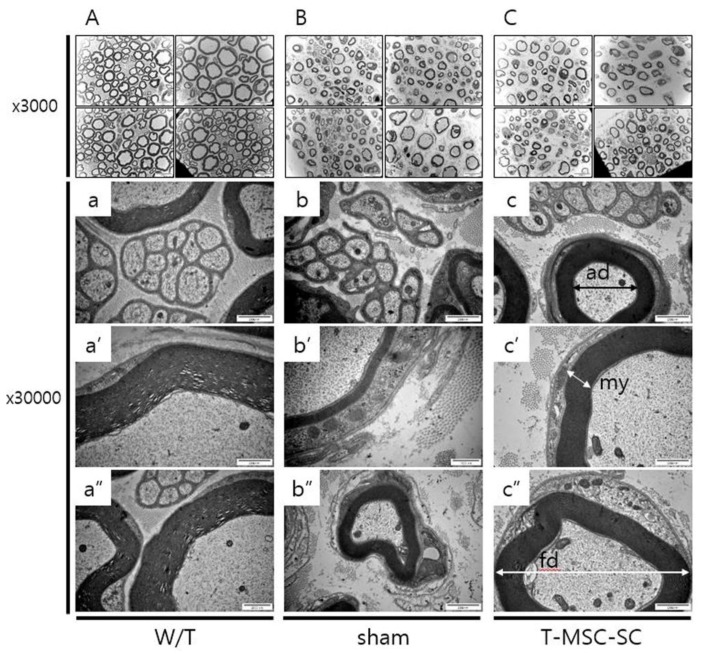
Assessment of ultrastructure of sciatic nerves in Tr-J mice following transplantation with T-MSC-SCs by electron microscopy (EM). (**A**–**C**): ×3000 magnification; (**a**–**c’’**): ×30,000 magnification. Relative to wild-type (W/T) sciatic nerve, the myelinated nerve axon and large diameter axon were thinner in Tr-J mice than those in the sham and T-MSC-SC groups. ad (axonal diameter); fd (fiber diameter); my (myelin thickness).

To examine whether transplanting T-MSC-SCs could affect remyelination in Tr-J mice, we analyzed the sciatic nerve using electron microscopy (EM). The most representative photographs of each experimental field are shown in Figure 4. In Tr-J mice, the ratio of myelin thickness (my) surrounding axons was reduced compared with W/T mice (Figure 4a–a’’). However, the ratios of my in mice from the T-MSC-SC group were elevated compared with sham mice (Figure 4b–c’’). The amount of myelin sheath (fiber diameter, fd) increases in proportion to the axon diameter (ad) [25]. The ratio of myelinated fibers increased in T-MSC-SC group mice (68.87%) compared with the sham group (56.89%), although it was markedly higher in the W/T group mice (80.0%) than in either of the other groups (sham and T-MSC-SCs). EM showed enhancement of remyelination in Tr-J mice after T-MSC-SCs transplantation.

### 2.4. Western Blot Analysis of the Sciatic Nerve

**Figure 5:**
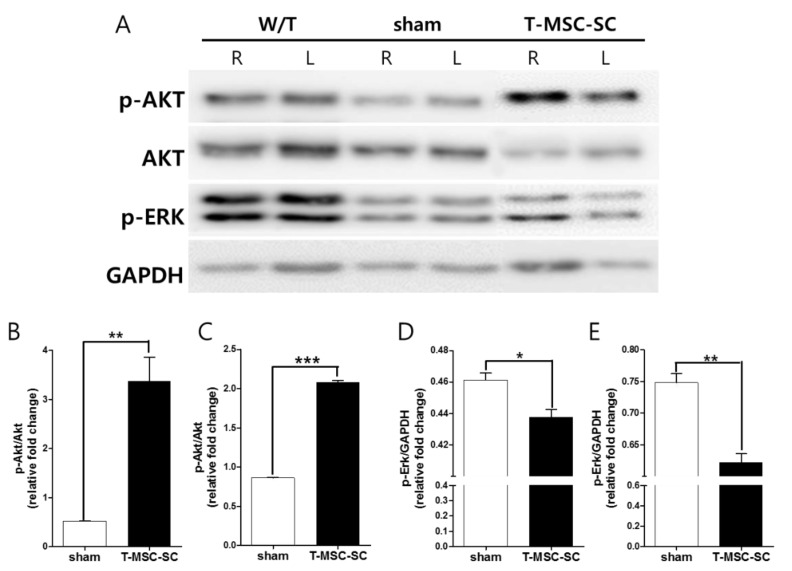
Effect on sciatic nerve regeneration by T-MSC-SCs suggested by expression of PI3K–Akt and Mer–Erk signaling pathways in Tr-J mice. Western blotting (**A**) and respective quantifications showing Akt activation (as measured by phosphorylation, p-Akt) and Erk expression (as measured by GAPDH) in right and left sciatic nerve lysates from Tr-J and W/T mice at 12 weeks after injection (**B**: Akt activation in right; **C**: Akt activation in left; **D**: Erk expression in right; **E**: Erk expression in left). The levels of GAPDH were measured as a loading control. Band intensities were quantified using ImageJ software. Data are the means ± SEM of experiments performed in triplicate (*n* = 3; * *p* < 0.05; ** *p* < 0.01; *** *p* < 0.001). R, right; L, left.

We then examined whether transplantation of the T-MSC-SCs was associated with sciatic nerve SC regeneration in Tr-J mice. Western blot analysis (Figure 5) of the phosphatidylinositol 4,5-bisphosphate 3-kinase (PI3K)-v-Akt murine thymoma viral oncogene homolog 1 (Akt) and the mitogen-activated protein kinase 1 (Mek)-mitogen-activated protein kinase (Erk) signaling pathways displayed greater induction of PI3K–Akt signaling in mice in the T-MSC-SC group than mice in the sham group (Figure 5B right and Figure 5C left). By contrast, the Erk expression ratio in mice in the sham group was higher than that in mice in the T-MSC-SC group (Figure 5D right and Figure 5E (left), which suggests an imbalance between PI3K–Akt and Mer–Erk signaling caused by transplantation of T-MSC-SCs. Interestingly, the T-MSC-SCs were implanted only in the right leg, but a similar pattern of signal pathway of myelination was observed in the nerves of both legs.

### 2.5. Expression of NF-H and MBP by Immunohistochemistry

**Figure 6:**
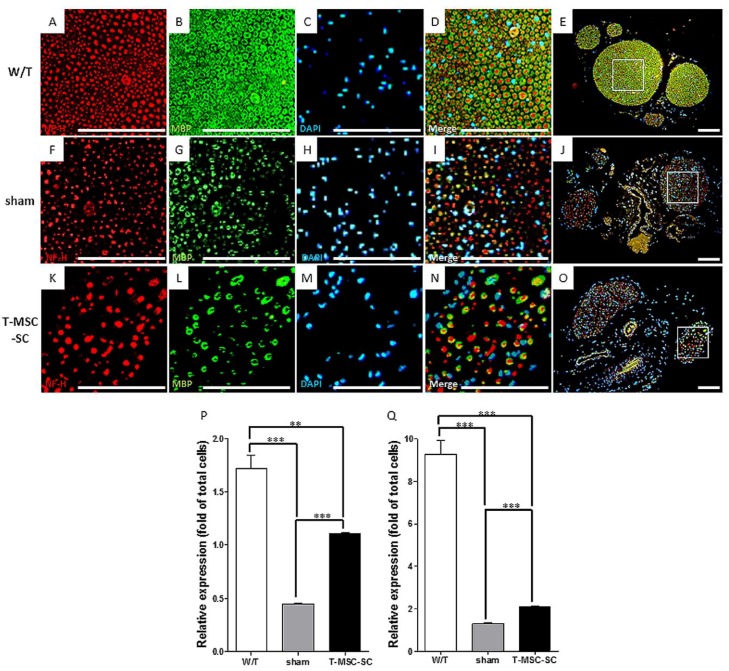
Remyelination and sciatic nerve regeneration by T-MSC-SCs transplanted into Tr-J mice. T-MSC-SC (**K**–**O**) or control (phosphate-buffered saline (PBS)) alone (sham; **F**–**J**) were injected into the thigh muscle. Age-matched W/T animals (**A**–**E**) were used as normal controls. The biopsy specimens obtained at 12 weeks after injection. Representative images of the sciatic nerve regeneration showing neurofilament-H (NF-H; red) and the remyelination showing myelin basic protein (MBP; green), and DAPI (blue) staining on a sciatic nerve cross-section from the W/T, sham, and TMSC-SC groups. Boxed areas in **E**, **J**, and **O** are enlarged in **D**, **I**, and **N**, respectively. Scale bars = 100 μm. Fluorescence intensities were quantified using ImageJ software (**P**, NF-H; **Q**, MBP). Data are the means ± SEM of experiments performed in triplicate. ** *p* < 0.01, *** *p* < 0.001.

Notably, in Tr-J mice, immunohistochemistry (IHC) revealed remyelination and nerve regeneration in mice in the T-MSC-SC group (Figure 6A–O). Although not as ideal as the nerves from mice in the W/T group (Figure 6A–E), the sciatic nerves from mice in the T-MSC-SC group (Figure 6K–O) revealed regenerative morphology in the axons surrounded by myelin sheaths, with enhanced expression of neurofilament-H (NF-H) (Figure 6K) and myelin basic protein (MBP) (Figure 6L), compared with the sham group (Figure 5F–J), in which numerous axons (Figure 6F) and myelin sheaths (Figure 6G) were destroyed. The regeneration was quantified by the fluorescence intensities of NF-H (Figure 6P, W/T: 1.72 ± 0.122; sham: 0.44 ± 0.012; T-MSC-SC: 1.11 ± 0.005) and MBP (Figure 6Q, W/T: 9.27 ± 0.653; sham: 1.29 ± 0.035; transplantation: 2.07 ± 0.076) in the stained sciatic nerves, as well as changes in images.

### 2.6. Skeletal Muscle Regeneration after T-MSC-SCs Transplantation

**Figure 7:**
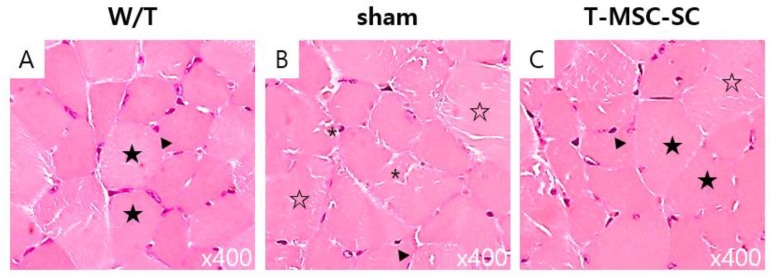
Skeletal muscle regeneration in Tr-J mice with transplanted T-MSC-SCs. T-MSCs-SCs and/or phosphate-buffered saline (sham) was transplanted into the right thigh muscle near the sciatic nerve in Tr-J mice. Age-matched wild-type (W/T) mice were used as normal controls. The biopsy specimens were obtained at 12 weeks after intervention. Cross-sections of gastrocnemius muscle were stained with HE. Original magnification ×400. * Small rounded fiber; ▼ nuclei; ☆ big rounded fiber; ★ polygonal shape.

**Figure 8:**
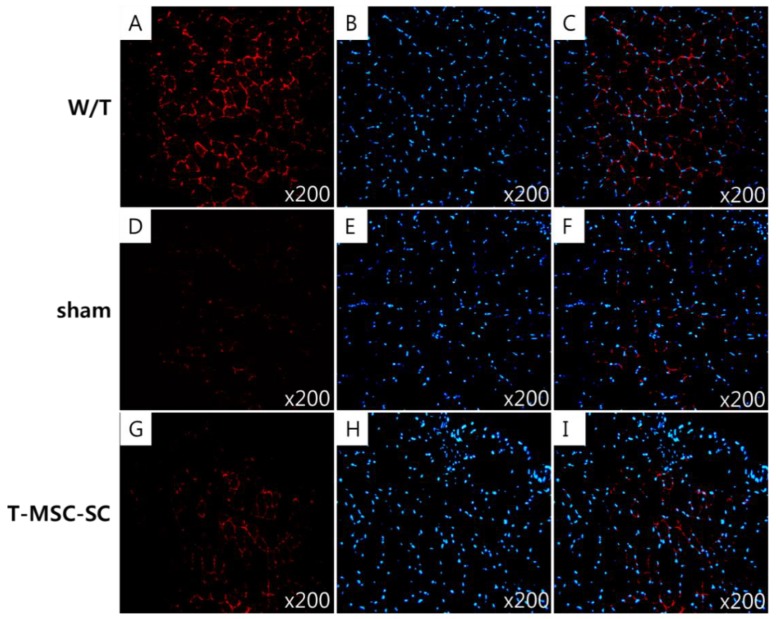
Dystrophin immunoreactivity in Tr-J mice with transplanted T-MSC-SCs. Age-matched wild-type (W/T) mice were used as normal controls. The biopsy specimens were obtained at 12 weeks after transplantation. Cross-sections of gastrocnemius muscle immunostained for dystrophin (red; **A**,**D**,**G**) and stained with DAPI (blue; **B**,**E**,**H**), merged images show dystrophin and DAPI (**C**,**F**,**I**). Original magnification ×200.

Following the remyelination and nerve regeneration in Tr-J mice with transplanted T-MSC-SCs, we examined skeletal muscle regeneration using hematoxylin and eosin (HE) staining and IHC. HE staining showed that normal myofibers in W/T mice have a polygonal shape, an intact sarcolemma, nonfragmented sarcoplasm, and homogeneous size distribution (Figure 7A). The muscle fibers of Tr-J mice (Figure 7B,C) showed lighter staining than the intact fibers of W/T mice, irregular muscle fiber shape, and small or very big and rounded fibers. Further, fewer nuclei appeared in mice in the sham group (Figure 7B) than in the T-MSC-SC and W/T groups. Notably, there was an improvement in the amount of intact sarcolemma, which showed slightly decreased and homogeneous size in mice in the T-MSC-SC group (Figure 7C) than in mice in the sham group. We observed the expression of dystrophin (red) in the gastrocnemius of Tr-J mice, and mice in the W/T, sham, and T-MSC-SC groups at 12 weeks after transplantation. Dystrophin is a protein located between the sarcolemmas that supports muscle fiber strength, and the absence of dystrophin reduces muscle stiffness [26]. Although less than that in W/T mice (Figure 8A–C), there was more definite expression of dystrophin in the endomysium of mice in the T-MSC-SC group (Figure 8G–I) than in mice in the sham group (Figure 8D–F), where dystrophin immunoreactivity was only faint. These results suggest that the remyelination and nerve regeneration by the transplantation of T-MSC-SCs into Tr-J mice can lead to skeletal muscle regeneration.

## 3. Discussion

The T-MSCs used in this study have previously been reported capable of cell banking with immunomodulatory activity for clinical use. As tonsillar tissues are discarded after surgery, the isolation of stem cells from these discarded tissues offers another valuable means of recycling human tissue for stem cell therapy [20,23]. In addition, the T-MSCs have MSC characteristics and are known to secrete various cytokines [27], growth factors [28], and exosomes [29] considered suitable for cell therapy. The T-MSCs show excellent immune modulation activity and no immunologic problems even when the T-MSCs were combined from three independent donors [23]. Thus, we speculated that the T-MSCs would be suitable for clinical use as a heterologous transplantation cell source.

In this study, we observed the beneficial effects of the transplantation of T-MSC-SCs that were confirmed by the increased expression of Schwann cell (SC) markers, including S100, *NGFR*, *GFAP*, *BDNF*, and *GDNF*. When T-MSC-SCs were transplanted into the caudal thigh muscles of Tr-J mice, amelioration of sciatic nerve demyelination and skeletal muscle regeneration were observed in T-MSC-SCs recipient mice. The therapeutic targets in demyelinating forms of CMT1A are those arising from a duplication of the gene encoding PMP22 in demyelinating SCs [30]. The improvement of remyelination after transplantation of T-MSC-SCs in Tr-J mice was suggested by the increased myelin thickness and large axons by EM, and the upregulation of MBP by immunostaining of nerves. Western blotting of proteins in the PI3K–Akt and Mek–Erk pathways showed induction of PI3K–Akt signaling and reduction of Mek–Erk signaling in sciatic nerve from Tr-J mice injected with T-MSC-SCs 12 weeks after transplantation. These results are consistent with reports of decreased activation of the PI3K–Akt pathway and increased activation of the Mek–Erk pathway in peripheral nerves of Pmp22-overexpressing rat models of CMT [31] and the disrupted maturation of SCs because of an imbalance between the PI3K–Akt and Mek–Erk pathways that contributes to the pathogenesis of CMT1A [32].

The clinical phenotype of CMT is one of neurogenic muscle atrophy [2.3]. In particular, a point mutation in *pmp22* of inbred Tr-J mice as an animal model of CMT1A leads to peripheral nervous system and neuromuscular dysfunction [33]. The immunostaining results of the present study suggest the pattern of MBP and NF-H in the regenerating sciatic nerves in mice from the group with transplantation of T-MSC-SCs, which indicated that the differentiated SCs facilitated remyelination and axonal regrowth. Tr-J mice in the group with transplantation of T-MSC-SCs showed larger axons and myelin sheaths than mice in the sham group. Transplantation of T-MSC-SCs clearly enhanced sciatic nerve regeneration by restoring the myelin sheath and axons of peripheral nerves in the Tr-J mice. We examined how transplantation with T-MSC-SCs influenced behavior and regeneration of skeletal muscle, and established that the transplantation reversed muscle atrophy by regenerating and restoring myofibers in the Tr-J mice. During the 12-week behavioral studies, we observed that the abnormal gait seen as tremor and irregular stride of Tr-J mice improved after transplantation with T-MSC-SC using SFI and that the T-MSC-SCs-recipient mice were able to stand with their front limbs supported on a wall (Figure S1). Furthermore, there was increased dystrophin immunoreactivity in the skeletal muscle in mice in the group receiving the T-MSC-SCs transplantation. These results suggest that T-MSC-SCs promote nerve and skeletal muscle regeneration. Similar to this study, Ad-MSC-derived SCs could also reduce muscle atrophy and enable nerve repair, and BM-MSCs could promote the regeneration of peripheral nerves after intramuscular transplantation [34,35]. From a different viewpoint, the transplantation of T-MSC-derived myocytes into the gastrocnemius of Tr-J mice have confirmed the improvement in motor function and the recovery of sciatic nerve and skeletal muscle seen in our previous study [36]. The regeneration of peripheral nerves and adjacent muscles seems to be highly correlated. To make the transplantation of T-MSC-SCs applicable for clinical purposes, further studies are needed; these include determination of the survival rate and migration route of the transplanted cells, in addition to safety studies.

The T-MSC-SC effected the potentially therapeutic action of remyelination and regeneration of the sciatic nerve and the skeletal muscle after they were transplanted into muscle in the present study. We propose that the therapeutic actions for peripheral neuropathy in Tr-J mice shown in the present study were from the paracrine effects of transplanted T-MSC-SCs. Several articles contend that the stem-cell treatment effects in animal models of peripheral neuropathy are paracrine effects without identifying a clear therapeutic mechanism. Neurotrophic factors have been reported as having paracrine effects and as the primary factors for peripheral nerve regeneration including promotion of axonal growth and remyelination [6]. The increase of neurotrophic factors appears not only to mediate the process of differentiation to SCs but also the regeneration of tissue after stem-cell transplantation. The MSC-derived exosome is noticed to have potentially therapeutic effectors, and includes cytokines and growth factors, signaling lipids, mRNAs, and regulatory miRNAs [32]. Therefore, exome treatments for various diseases have been increasingly reported. Based on these reports, neurotrophic factors and epidermal growth factor-like protein neuregulin-1, a physiological activator of PI3K–Akt signaling, which are components of the exosome, can be assumed to be the mediators of the sciatic nerve and skeletal muscle regeneration in the present study. Further studies such as components analysis of exosome derived from T-MSC-SCs and application of exosomes for the treatment of Tr-J mice should be needed. In addition, the Tr-J mouse is not the best model and is thus not optimal for human application to study CMT1A. The C3 mouse, an authentic model of the *PMP22* duplication, is available [37]. Application of cell therapy to the C3 mouse will provide more precise information for the clinical application of cell therapy for patients with CMT1A.

## 4. Material and Methods

### 4.1. Ethics Statement

The T-MSCs were derived from patients undergoing tonsillectomy. The Institutional Review Board of Ewha Womans University, Mokdong Hospital (Seoul, Korea) approved all the experimental procedures used in this study (permit No. ECT-11-53-02; approval date: 22, September, 2011). Informed written consent was secured from all patients and/or their legal representatives before obtaining tonsillar tissues. The use and care of experimental animals were performed according to the guidelines of the Korean Ministry of Health and Welfare and were approved by the Institutional Animal Care and Use Committee at Ewha Womans University School of Medicine (permit No. ESM-15-0315; approval date: 1, September, 2015). All experiments were performed in accordance with the approved guidelines and regulations of the Animal Care Guidelines of the Ewha Womans University, and the Guide for the Care and Use of Laboratory Animals [38].

### 4.2. Animals

The Tr-J mutation introduces a novel *Ban*I restriction site in the *pmp22* sequence [39]. Heterozygous Tr-J male mice from a C57BL/6J black mouse background strain were purchased from Jackson Laboratories (Bar Harbor, ME, USA) and were used to establish a breeding colony at Samsung Medical Center. Eight-week-old mice were housed at 21 ± 2 °C and 55 ± 5% humidity with a 12/12-h light/dark cycle and were supplied with autoclaved food and water ad libitum. The mice used in the present study were 35 days old and underwent a phenotypic evaluation called the tail suspension test (TST) [40,41]. W/T mice fully extended the hind limb phalanges and maintained this extension, but all the heterozygous Tr-J mice performed frequent abnormal flexion–extension movements during the TST accompanied by a subsequent flexion of the posterior phalanges (Figure 2A). For the present study, the animals were randomized, and the rotarod test, gait test, EM, Western blot analysis, and immunohistochemical staining were performed with the observers blinded to the treatment modality (Figure 2C).

### 4.3. Preparation of T-MSCs and Differentiation into Schwann Cells

T-MSCs were isolated from tonsils collected from patients during tonsillectomy, as previously described [19,21,42]. We isolated four different T-MSC lines from each of four patients. The excised tonsils were minced and digested in Dulbecco’s modified Eagle’s medium (DMEM; Invitrogen, Carlsbad, CA, USA) containing 210 U/mL collagenase type I (Invitrogen) and DNase (10 μg/mL; Sigma-Aldrich, St. Louis, MO, USA). After the cells had been passed through a cell strainer (BD Biosciences, San Jose, CA, USA), mononuclear cells were obtained by Ficoll-Paque density gradient centrifugation (GE Healthcare, Chicago, IL, USA). The cells were cultured for 48 h at 37 °C in low-glucose DMEM containing 10% fetal bovine serum (FBS; Invitrogen) and 1% penicillin/streptomycin (Sigma-Aldrich) in a humidified chamber under 5% CO_2_ in air. To induce SC differentiation of T-MSCs, 1.5–2 × 10^5^ cells/cm^2^ cells were plated in a plastic dish in DMEM/F-12 (Invitrogen) supplemented with 20 ng/mL basic fibroblast growth factor (bFGF, PeproTech, London, UK), 20 ng/mL epidermal growth factor (PeproTech), and 2% B27 supplement (1:50, Gibco, Life Technologies, Burlington, ON, Canada) at 37 °C under 5% CO_2_ in humidified air. After 7 days, cells aggregated spontaneously to form neurospheres that were triturated using a 25-gauge needle and plated in laminin-coated cell culture plates containing DMEM/F12 supplemented with 10% FBS, 14 μM forskolin (Sigma-Aldrich), 5 ng/mL platelet-derived growth factor-AA (PeproTech), 10 ng/mL bFGF (PeproTech), and 200 ng/mL recombinant human heregulin-β1 (PeproTech) for terminal differentiation. The cells were differentiated for 9 days under these conditions, and then harvested for characterization and transplantation.

### 4.4. Immunocytochemistry

The cells were grown on coverslips fixed in 4% (*v*/*v*) PFA (Sigma-Aldrich) for 15 min at room temperature, or overnight at 4 °C. After rinsing in PBS, the fixed cells were permeabilized and nonspecific epitopes were blocked using 2% bovine serum albumin (Bovogen Biologicals, East Keilor, VIC, Australia) in 0.1% Tween-20/PBS, followed by incubation in the diluted primary antibody for 1 h at room temperature, or overnight at 4 °C, with an anti-GFAP monoclonal antibody (1:200, Sigma-Aldrich, cat. no. G3893) or an anti-NGFR polyclonal antibody (1:200, Santa Cruz Biotechnology, cat. no. sc8317). Following three washes in PBS, the samples were incubated for 1 h at room temperature with secondary antibodies diluted in PBS. The prepared samples were then mounted using Vectashield mounting medium containing 4',6-diamidino-2-phenylindole (DAPI; Vector Laboratories, Burlingame, CA, USA) and images were captured under a fluorescence microscope (Nikon Corp., Tokyo, Japan).

### 4.5. Real-Time Quantitative Polymerase Chain Reaction (Real-Time qPCR)

Total RNA was extracted from cells using Qiagen RNeasy Mini Kits (Qiagen, Hilden, Germany). Complementary DNA (cDNA) was synthesized using Superscript II (Invitrogen) and oligo(dT)20 primers at 42 °C for 1 h followed by incubation at 72 °C for 15 min. Real-time qPCR was performed using SYBR Premix Ex Taq DNA polymerase (TaKaRa Bio Inc., Shiga, Japan) on an ABI 7500 Fast Real-time PCR system (PE Applied Biosystems, Foster City, CA, USA) to confirm the relative levels of expression of genes in the T-MSCs and T-MS-SCs. The total volume of the PCR reaction was 20 μL, containing 0.8 μL of each primer (5 μM), 1 μL cDNA, 10 μL 2× SYBR Premix Ex Taq II DNA polymerase, 0.4 μL ROX dye, and 7.8 μL sterile double-distilled H_2_O. PCR cycling conditions were as follows: initial 30 s denaturation at 95 °C, followed by 40 cycles of amplification at 95 °C for 3 s, 60 °C for 30 s, and a subsequent melting curve analysis, where the temperature was increased from 60 to 95 °C. To quantify the expression of each candidate gene, the mRNA expression levels were normalized to the level of glyceraldehyde 3-phosphate dehydrogenase (*GAPDH*) mRNA. Relative gene expression was analyzed using a comparative cycle threshold (*C*_t_) method (ΔΔ*C*_t_) [43]. Real-time qPCR was performed in triplicate for each sample and was repeated three times for each assay. The sequences of forward and reverse primers used were as follows: *S100B* forward 5′-GGAGACGGCGAATGTGACTT-3′, reverse 5′-GAACTCGTGGCAGGCAGTAGTAA-3′; *GFAP* forward 5′-CCGACAGCAGGTCCATGTG-3′, reverse 5′-GTTGCTGGACGCCATTGC-3′; *NGFR* forward 5′-CCTACGGCTACTACCAGGAT-3′, reverse 5′-TGGCCTCGTCGGAATACG-3′; *GDNF* forward 5′-TTCAAGCCACCATTAAAAGAC-3′, reverse 5′-GACAAAGGTGTGAGTCGTGGT-3′; *BDNF*, forward 5′-GATGCCAGTTGCTTTGTCTTC-3′, reverse 5′-TAAAATCTCGTCTCCCCAACA-3′; *GAPDH*, forward 5′-ACACCCACTCCTCCACCTTT-3′, reverse 5′-TGCTGTAGCCAAATTCGTTG-3′.

### 4.6. Transplantation

Transplantation was performed with the mouse under general anesthesia effected with a mixture of Zoletil 50 (Virbac, Carros, France) and Rompun (Bayer Korea, Seoul, Republic of Korea), 3:1 ratio, 1 mL/kg intraperitoneally. At 6 weeks after birth, 1 × 10^6^ T-MSC-SCs in 100 μL PBS (T-MSC-SC-injected group (*n* = 7), T-MSC-SC) or 100 μL PBS alone (sham vehicle-treated group (*n* = 7), sham) was injected intramuscularly into the right thigh muscle near the sciatic nerve (Figure 2B). Age-matched wild-type mice (no transplantation (*n* = 7), W/T) and Tr-J mice were studied using the rotarod behavior test (below) at 2, 4, 6, 8, 10, and 12 weeks, and the SFI measured at 12 weeks. Finally, all mice were humanely killed to obtain tissues for EM and immunohistochemistry at 12 weeks after the interventions (Figure 2C).

### 4.7. Rotarod Test

To evaluate the motor coordination and balance of Tr-J mice, they were placed on a 3 cm horizontal rotating rod (2 m/min) after being pretrained for 7 days. The test comprised three trials separated by 15 min intertrial intervals, and the testing was done for a maximum of 7 min.

### 4.8. Sciatic Functional Index (SFI)

The bottom of each hindfoot of each mouse was coated with nontoxic ink, and the mouse was allowed to walk in a straight line along an 80 cm long runway over paper. The footprint patterns were then observed. A series of at least five sequential steps recorded in the same session was used to determine the walking pattern for each mouse. Footprints were analyzed quantitatively with the gait test and evaluation of SFI, as described previously [44]. The parameters of toe spread and paw-print length from the intact and injured or transplanted sides were assessed to calculate the SFI.

### 4.9. Electron Microscopy

For the ultrastructural observations, the right sciatic nerve tissue was fixed with 2% glutaraldehyde in 0.025 M cacodylate buffer at pH 7.4, post-fixed in 1% osmium tetroxide, followed by embedding in Epon using a standard procedure. Epon-embedded blocks were cut at 80 nm with a diamond knife. The ultrathin sections were double-stained with uranyl acetate and lead citrate for electron microscopy. The same block faces were cut at 1 μm with a sapphire knife replacing a diamond knife. These semithin sections were fixed onto lysine-coated slide glasses laid on a hot plate at 60–70 °C. Ultrathin sections were prepared using a Leica ultratome (Reichert Ultracuts, Wien, Austria) and stained with 4% uranyl acetate for 45 min, and subsequently with lead citrate for 4 min at room temperature. Sections were examined using an H-7650 electron microscope (Hitachi, Ibaraki-ken, Japan).

### 4.10. Western Blotting

Sciatic nerve tissues were washed with ice-cold PBS and lysed in Pro-Prep buffer containing a phosphatase inhibitor cocktail solution (iNtRON Biotechnology, Seongnam-si, Korea) for 30 min on ice. After centrifugation at 13,000× *g* for 20 min at 4 °C, equal quantities of protein from supernatants were separated by sodium dodecyl sulfate–polyacrylamide gel electrophoresis (SDS–PAGE) and were electrophoretically transferred onto polyvinylidene membranes (Millipore, Billerica, MA, USA). The blots were then probed overnight at 4 °C with antibody against v-Akt Murine Thymoma Viral Oncogene (Akt; No. 9272) or Phospho-Akt (p-Akt; Thr308; No. 9275) or extracellular-signal-regulated kinase (ERK; Phospho-p44/43 MAPK (Erk1/2) (Thr202/Tyr204); No. 9101) (1:500, all pRb, Cell signaling) followed by the corresponding secondary antibody. The blots were washed and developed using enhanced chemiluminescence reagents (WestSave Gold Western Blot Detection kits; AbFrontier, Seoul, Korea), according to the manufacturer’s instructions. Band intensities were assessed by densitometric scanning (LAS-3000, Fujifilm, Tokyo, Japan). The p-Erk protein expression level was normalized to the expression of GAPDH (No. LF-PA0018; 1:1000, pRb, Ab Frontier, Korea). The results were quantified using Multi Gauge software, version 3.0.

### 4.11. Immunohistochemistry and HE Staining

The mouse sciatic nerves and gastrocnemius muscles were fixed in 10% formaldehyde. Following approximately 24 h of fixation at 4 °C, the nerves and muscles were washed in PBS at room temperature. The washed tissues were dehydrated in a graded series of ethanol concentrations, cleared in xylene, and embedded in paraffin wax. The embedded tissues were sectioned into 5-μm-thick serial sections and placed onto microscope slides for staining. Nonspecific epitopes were blocked with 10% normal goat serum albumin in 0.1% Triton X-100/PBS followed by incubation with the appropriate primary antibody for 1 h at room temperature. After three washes in 0.01% Triton X-100/PBS, the sections were incubated with secondary antibodies for 1 h at room temperature, or at 4 °C overnight. The stained tissues were mounted using Vectashield medium containing DAPI (Vector Laboratories) and photographed using a fluorescence microscope (Nikon Corporation, Japan). The manufacturers and catalog numbers (Cat. No.) of the antibodies employed are as follows: rabbit anti-myelin basic protein (MBP; Merck Millipore, Billerica, MA, USA; Cat. No. AB980), mouse anti-neurofilament heavy polypeptide (NF-H; Santa Cruz Biotechnology Inc., Dallas TX, USA; Cat. No. sc-58553), rabbit anti-dystrophin (Abcam, Cat. No. ab-15277), Alexa-568-conjugated goat anti-rabbit IgG (Life Technologies, Cat. No. A-11057), and Alexa-488-conjugated goat anti-mouse IgG (Life Technologies, Cat. No A-11001). For HE staining, sections of 5 μm thickness were cut and stained with a standard technique to determine the histological changes in the three groups. The stained slides were observed and photographed under a light microscope (Nikon Corporation, Tokyo, Japan).

### 4.12. Statistical Analysis

The results are presented as the mean ± standard error of the mean (SEM). Statistical comparisons were analyzed using Student’s *t*-tests for comparisons between two groups and by one-way analysis of variance (ANOVA) for the three groups using GraphPad Prism software (5.01; GraphPad Software Inc., San Diego, CA, USA) to identify significant differences. A *p* < 0.05 was considered significant. All the experiments were performed at least three times, and all animals performed a rotarod test, and SFI and EM observations were made by observers blinded to the treatment group.

## 5. Conclusions

Here, we obtained transplantation of the T-MSC-SCs into the thigh muscle in Tr-J mice resulting in functional remyelination and significantly accelerated regeneration of the sciatic nerve and the skeletal muscle. Adverse effects, including teratoma formation in transplantation sites, were not observed. Further studies such as the assessment of neurotrophic releasing effects and regeneration of neuromuscular junctions might provide for clinical application in peripheral neuropathy. The differentiated SCs from human T-MSC may be applicable for novel regeneration therapy, not only for Charcot-Marie-Tooth disease 1A, but also for other peripheral neuropathies related to SC dysfunction.

## Supplementary Materials

Materials can be found at http://www.mdpi.com/1422-0067/19/8/2393/s1.

## Abbreviations

CMT1A	Charcot-Marie-Tooth type1A
T-MSC	tonsil-derived stem cell
Tr-J	trembler-J
SC	Schwann cell
T-MSC-SC	T-MSC differentiation toward the SC
PMP22	peripheral myelin protein 22
ESC	embryonic stem cell
iPSC	induced pluripotent stem cell
BM-MSCs	bone marrow-derived stem cells
Ad-MSC	adipose-derived Stem cells
UC-MSCs	umbilical cord-derived MSCs
SFI	sciatic function index
EM	electron microscopy
TST	tail suspension test
