# Supplementary material for: Differentiation of Human Tonsil-Derived Mesenchymal Stem Cells into Schwann-Like Cells Improves Neuromuscular Function in a Mouse Model of Charcot-Marie-Tooth Disease Type 1A
Source: Int J Mol Sci. 2018 Aug 14;19(8):2393. doi: 10.3390/ijms19082393 (PMC6121309; doi:10.3390/ijms19082393)
Supplement: Supplementary file 1 [file ijms-19-02393-s001.zip › ijms-325523-SI.pdf]

Supplementary materials

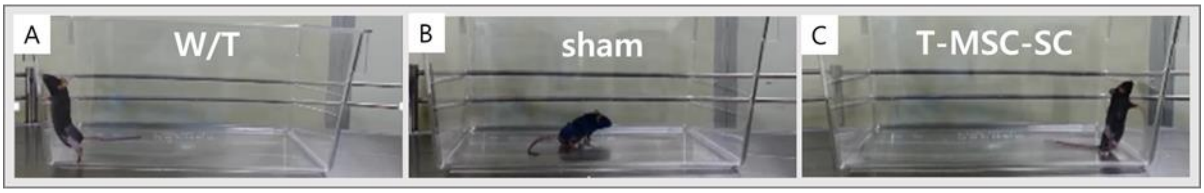

Figure S1. Restoration by the transplantation of Schwann cells differentiated from human T-MSCs into trembler-J (Tr-J) mice. Some 12 weeks after transplantation of T-MSC-SC, the Tr-J mice became stronger on their hind legs and they could stand up with their forelimbs holding the wall of their cage (**C**) as the wild-type mouse could (**A**). There was no change in the sham group mice (**B**) at 12 weeks after they were injected with PBS.
